# Maternal Influenza and Offspring Neurodevelopment

**DOI:** 10.3390/cimb46010023

**Published:** 2024-01-02

**Authors:** Marya Egorova, Vladimir Egorov, Yana Zabrodskaya

**Affiliations:** 1 Smorodintsev Research Institute of Influenza, Russian Ministry of Health, 15/17 Ulitsa Prof. Popova, St. Petersburg 197376, Russia; sci-work_maria@mail.ru (M.E.); sondyn@yandex.ru (V.E.); 2 Institute of Experimental Medicine, 12 Ulitsa Akademika Pavlova, St. Petersburg 197376, Russia; 3 Institute of Biomedical Systems and Biotechnology, Peter the Great Saint Petersburg Polytechnic University, 29 Ulitsa Polytechnicheskaya, St. Petersburg 194064, Russia

**Keywords:** maternal influenza infection, immune system, neurodevelopment, neuropsychiatric disorders, animal models, infectious diseases, pregnancy

## Abstract

This review examines the complex interactions between maternal influenza infection, the immune system, and the neurodevelopment of the offspring. It highlights the importance of high-quality studies to clarify the association between maternal exposure to the virus and neuropsychiatric disorders in the offspring. Additionally, it emphasizes that the development of accurate animal models is vital for studying the impact of infectious diseases during pregnancy and identifying potential therapeutic targets. By drawing attention to the complex nature of these interactions, this review underscores the need for ongoing research to improve the understanding and outcomes for pregnant women and their offspring.

## 1. Background

Influenza epidemics and pandemics represent a major health problem worldwide. Groups at risk for complications from seasonal influenza include certain age groups (persons under 2 and over 65 years), people with chronic diseases (chronic lung disease, neurological disorders, diabetes), and pregnant women [1]. Influenza infection during the first trimester of pregnancy has been associated with a 2.0-fold increased risk of any major malformation, a 3.3-times increased risk of neural tube defects, and a 1.6-times increased risk of congenital heart defects [2]. It is important to note that a study by Kim Newsome and colleagues at the National CDC following the 2009 influenza A (H1N1) pandemic found that severely ill women with 2009 H1N1 influenza during pregnancy were more likely to experience adverse birth outcomes, such as preterm birth or low birth weight [3]. Influenza A virus (IAV) infection during pregnancy not only leads to serious morbidity and mortality in pregnant women but also increases the risk of adverse fetal outcomes, including preterm birth, low birth weight, and even infant mortality [4].

It is indeed true that the mechanisms of the affection of maternal influenza infection on the fetus have not been thoroughly studied [5]. Some suggestions have been made that the effects on the fetus and pregnancy outcome may be an indirect result of maternal influenza infection, such as fever, cytokine, and immune responses [6]. Though viremia in influenza is thought to be uncommon [7], and placental transmission of the virus is rare [8], mouse models show that side effects can still occur, even in the absence of fetal virus infection. Prenatal influenza infection in mice has been associated with histopathological changes in the brain [9] and behavioral changes [10] in the offspring. Although influenza virus RNA has not been detected in the fetal brain, these changes suggest that the effects on the fetus may be secondary to the maternal inflammatory response rather than the result of direct viral exposure [5,11].

Stephen G. Fung’s et al. systematic review [12] examined the literature regarding the association between neurological complications in offspring and maternal influenza during pregnancy. The authors observed that, currently, there are limited and conflicting data on this topic. Some studies have suggested a potential association between maternal exposure to the influenza virus and the risk of developing schizophrenia [13,14], psychosis or psychosis-like experiences [15,16], mood disorders [17], developmental delays [17], attention deficit hyperactivity disorder (ADHD) [18], and bipolar disorder/bipolar affective disorder (with psychotic features) [19]. The results for schizophrenia were highly variable, and while three of the four studies reporting adjusted estimates were considered to have a low risk of bias [14,17,20], differences in reported estimates were attributed by the authors to differences in study methodology.

Several factors have been proposed to impact the severity and extent of potential fetal brain damage caused by maternal infection, including genetic predisposition, maternal trimester of illness, severity and duration of infection, and placental inflammation [21]. Recent findings suggest that inflammation resulting from maternal infections such as influenza may modify transcriptional programs in the placenta, consequently raising the risk of neurodevelopmental disorders, including schizophrenia, especially in the presence of a genetic predisposition [22], as well as increasing the severity of ASD symptoms [23].

The significance of genetic predisposition in evaluating the impact of prenatal environmental exposure on psychotic disorders has been underscored by many researchers [24]. A study [25] reported an increased risk of psychosis in the offspring of mothers with maternal infection during pregnancy in combination with a family history of mental illness. The influence of inflammatory gene interactions on psychosis-like behavior has also been reported in animal studies using mice [26,27].

Exposure of the fetus to maternal infection and inflammation during critical periods of development can lead to changes in brain structure and function [28]. A study using animal models found that exposure to maternal immune activation in early pregnancy was associated with changes in brain development mechanisms, leading to long-term consequences, including ASD; exposure late in pregnancy did not have the same effect [29].

Attention should be paid to viral infection-induced changes in the metabolic pathways of the maternal body, which can also affect the development of the fetal brain. Infection with influenza virus during pregnancy has been identified as a risk factor for neurodevelopmental disorders in the offspring, including the risk of developing schizophrenia, with the peak effect occurring in the 6th month of intrauterine development in humans and correlating with serologically proven infection in the mother. Exposure to influenza A virus during development results in a transient increase in kynurenic acid concentrations, which can disrupt normal brain development and lead to cognitive impairment later in life. This may support understanding of how virus-induced changes in tryptophan metabolism during development may contribute to the onset of schizophrenia-related symptoms later in life [30].

Animal studies describing the role of maternal immune activation in neuropsychiatric illness have found changes in dopaminergic and GABAergic activity in the fetal brain, which have been similarly confirmed in human studies studying schizophrenia and ASD [31,32,33,34,35,36].

It is also worth mentioning a factor that is not considered in a number of studies and is associated with the course of influenza in humans. Medicines such as analgesics and anti-inflammatory drugs taken for infections during pregnancy may affect the development of the fetal nervous system [37]. The authors emphasize the insufficient understanding of the impact of fever and pain medications on fetal brain development. Although the use of antipyretic and analgesic medications during pregnancy may influence the maternal immune response and fetal brain development, most studies, including the ADHD study [18], have not considered the use of medications that could affect fetal brain development. However, studies have also reported a relation between fever during pregnancy and an increased risk of ASD [38,39] and ADHD [40]. Interestingly, it was found to be independent of adjustments for medication use.

**Figure 1:**
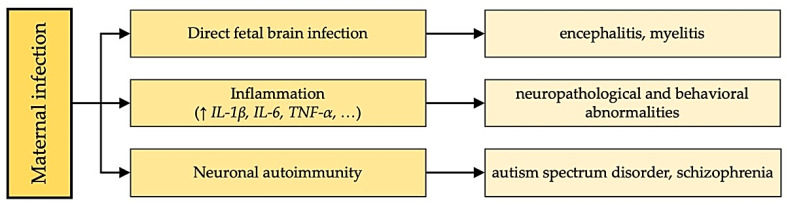
The possible mechanisms of occurrence of neurological complications during pregnancy influenza.

It is true that despite numerous studies, many molecular biological mechanisms of the occurrence of neurological complications during influenza infection remain unclear, and this problem does not lose its relevance. Research into the mechanisms of occurrence of neurological complications during influenza has been systematized into several areas [37] (Figure 1):

1. The live virus can directly enter the fetal brain and infect it. Interestingly, various researchers have obtained conflicting results. One group of researchers confirmed the presence of the virus in the fetal brain when the influenza virus was administered intranasally to pregnant mice [41]. Another study showed simultaneously that viral RNAs are not detectable in fetal mouse brains from infected mothers [11].
2. Chemical mediators of infection may mediate changes in brain development. During maternal infection, levels of chemical mediators of inflammation, primarily cytokines, interleukin-1β (IL-1β), IL-6, and tumor necrosis factor-α, increase in the maternal blood and placenta. Such cytokines can influence fetal brain development by directly affecting the fetal brain, interfering with placental function, or exerting effects through the mother. The fever-like state itself, because of the increased release of maternal cytokines, can affect the development of the fetal nervous system. Studies examining the risks associated with increased maternal body temperature have shown that even short-term exposure of pregnant rodents to high temperatures can lead to abnormalities in the central nervous system [42].
3. Autoimmune conditions: It has been suggested that immunoglobulins against infectious agents may cross-react with and damage fetal brain structures. There is no evidence yet that such an autoimmune mechanism occurs in schizophrenia, but the possibility of implementing such a mechanism is indirectly confirmed in some studies. For example, there is some experimental support for the idea that antibodies against group A β-hemolytic streptococci may cross-react with the basal ganglia, leading to cases of Tourette’s syndrome or obsessive-compulsive disorder following streptococcal throat infection in patients [43].

Possible mechanisms for the negative impact of influenza virus infection on prenatal and postnatal processes, which, if disrupted, can lead to an increased risk of developing schizophrenia or acute psychosis in adulthood, are summarized in a review [44].

## 2. Fetal Infection

The maternal organism has two types of protective systems to safeguard the fetus: mechanical and immunological defenses [45].

The evidence has indeed shown that IAV has been isolated from the placenta and amniotic fluid in both fatal [46,47] and non-fatal cases [48], although direct infection of the fetus has been rarely reported [49]. Placentitis caused by influenza virus infection is characterized by hyperplasia and the degeneration of amniotic cells, placental trophoblasts, decidual cells, and vascular endothelial cells. Viral antigens are also sometimes found in affected cells and in lymphoid cell infiltrates [50,51]. Additionally, studies have indicated that the human decidua provides a more favorable environment for virus replication than placental tissue. Influenza virus is able to spread from the maternal bloodstream into the maternal decidua, followed by tissue replication and infection of the fetal chorion and amnion [52], that could result from a direct cytopathic effect, potentially contributing to miscarriage associated with influenza virus infection [1]. Viremia resulting from IAV infection during pregnancy can lead to decidual and placental infections [46,48,53,54]. The data suggesting that the human decidua is the preferred environment for virus replication compared to placental tissue, and from there the virus spreads to the fetal membranes, are confirmed in the referenced studies [52,55]. These processes are likely responsible for adverse pregnancy outcomes associated with IAV infection [56].

It is true that small animal models (such as mice, ferrets) and pigs have been utilized in a number of studies to investigate complications associated with IAV during pregnancy. These experiments provide valuable insights into events early after infection and disease mechanisms that are challenging to study using materials from infected patients. Pregnant ferret and pig models may offer additional information about the pathogenicity of the virus observed in humans, although these models have been used in isolated studies to date. In general, ferrets and, to a lesser extent, pigs can mimic IAV-mediated respiratory pathogenesis, similar to that observed in humans [6].

Intranasal inoculation of seasonal H3N2 influenza virus into ferrets during early, mid, and late pregnancy resulted in viral replication in the respiratory tract comparable to that previously described for nonpregnant ferrets. However, the virus was not transmitted to the placenta, umbilical cord, amnion, and chorion of the fetus [57,58]. It is also noteworthy that the intracardiac inoculation of H3N2 virus resulted in transmission of the virus to the placenta and fetus, indicating that high-viral-load viremia may be critical for transmission of the virus to the fetus. It should be noted that, in general, in humans, seasonal influenza viruses do not cause viremia in nonpregnant women [59].

Intranasal or intratracheal inoculation with porcine H1N2, porcine H3N2, or A(H1N1)pdm09 influenza A virus of young pregnant pigs 85–90 days after insemination did not result in any clinical signs or gross pathological lesions 7 days after challenge. None of the experimental animals (*n* = 5 animals per group) showed signs of viremia or transplacental transmission of the virus [60,61]. However, another study found transplacental transmission of porcine H1N1 virus in one in ten pigs [62].

## 3. The Role of Cytokines

Influenza-induced increases in pro-inflammatory cytokines during pregnancy may alter the formation of critical fetal brain structures, which may play a role in the development of later neuropsychiatric diseases, as well as increased vulnerability to cerebral palsy and ASD [12]. Possible mechanisms by which viral infections may lead to autism include direct teratogenic effects and indirect effects of inflammation or maternal immune activation on the developing brain [63].

Interleukins (ILs) are a group of cytokines released in response to inflammatory processes and are potential candidates for influencing fetal brain development. Cytokines have the ability to cross the immature blood–brain barrier, and dysregulation of inflammatory cytokines such as IL-1, IL-6, and IL-17, as well as immunomodulatory cytokines like IL-2, has been observed in whole blood samples from patients with ASD, underscoring the significance of the immune response in the development of ASD [63]. Particularly, IL-6 is considered an indicator of maternal systemic inflammation, which may impact placental–fetal interactions and subsequent fetal brain development, thereby increasing the risk of neuropsychiatric disorders in the offspring [64]. These changes can lead to cognitive and behavioral deficits by affecting synapse formation and synaptic function in affected offspring [65]. The disruption of normal synaptic signaling and transmission can alter the balance of neurotransmitters and the number of excitatory and inhibitory connections in the developing brain, potentially leading to a wide range of adverse developmental outcomes [64].

A study by Ahmad Naqib Shuid et al. [63] indicates that increased levels of IL-6 during pregnancy may have the potential to alter brain architecture, executive function, and working memory capabilities in newborns. Since inflammatory markers, particularly IL-6, are expressed throughout the brain, it appears that cytokines can influence normal growth processes at each stage of fetal brain development.

Furthermore, viral infection can lead to the release of proinflammatory cytokines and activation of T-helper 17 cells in the maternal circulation [66,67]. Animal models of maternal immune activation suggest that the maternal inflammatory response may influence the early programming of various behaviors, including the ability to socialize and communicate, as well as the regulation of stereotypical behavior [63].

In human embryonic membranes in culture, infection with IAV induces apoptosis and gene expression of proinflammatory cytokines, such as IL-1β, IL-6, TNF-α, IFN-β, and -γ, as well as granulocyte-macrophage colony-stimulating factor, which may contribute in vivo to premature fetal membrane rupture due to the collapse of the amniotic epithelial cell layer [68]. It is indeed observed that influenza A virus (IAV) affects cultured chorionic cells from human fetal membrane tissues directly, leading to cytopathic effects such as cell detachment and rounding, as well as cellular degradation, such as oligonucleosomal DNA fragmentation and lactate dehydrogenase leakage in chorionic cells, which are characteristics of cells undergoing apoptosis [56,69]. These effects were not observed in infected, cultured amnion cells, which may result in persistent infection [56]. The synthesis of specific viral macromolecules at the early stage of infection plays a crucial role in inducing apoptosis [70,71]. Therefore, influenza A virus infection of the fetal chorionic membrane leads to a cellular proinflammatory cytokine response, with corresponding apoptosis [72].

Studies, involving several known inhibitors of cytokine production, suggest that cellular oxidation processes and peroxisome proliferator-activated receptor MAP kinase and NFk-B regulate the induction of proinflammatory cytokine gene expression in fetal membranes during infection [73,74,75]. This highlights the importance of balanced levels of pro-inflammatory and anti-inflammatory cytokines in controlling various intrauterine functions during infectious conditions, including influenza, during pregnancy [55].

In the model of experimentally infected pregnant gilts with swine influenza A virus strains H1N2, H3N2, and A(H1N1)pdm09, no evidence of transplacental transfer of IAV was found. At the same time, elevated serum levels of IL-6, IL-10, and TNF-α were observed. As a result, the authors [61] hypothesized that the main reason for pregnancy failure is related to the high fever and pro-inflammatory cytokines.

The role of hormones in the immune response to influenza during pregnancy is, indeed, complex and noteworthy. Progesterone and glucocorticoids, whose levels increase during pregnancy, may have anti-inflammatory effects [76]. Additionally, elevated progesterone levels may stimulate the synthesis of progesterone-induced binding factor, which promotes CD4+ T cell/T-helper type 2 (Th2) differentiation, leading to increased serum concentrations of Th2 cytokines, including IL-4, -5, and -10 [77,78,79]. This observed increase in Th2 responses during pregnancy corresponds to a decrease in Th1 responses both systemically and at the maternal–fetal interface in animal models as well as in humans [80,81,82,83,84,85]. Further investigation is needed to fully understand the direct role of progesterone in disease susceptibility and severity in the context of influenza infection.

The effect of estrogens on the severity of influenza infection is also complex, as elevated levels administered to nonpregnant mice are protective, while during pregnancy, they are not [86]. Estrogen appears to have both anti-inflammatory and pro-inflammatory effects [86,87]. The influenza infection seems to induce a hypoestrogenic state that affects these sodium channels, reducing alveolar fluid clearance and, thereby, increasing sensibility to pneumonia [88]. As a result, estrogen may influence disease severity through mechanisms unrelated to modulation of the immune system.

## 4. Autoimmune Conditions

During pregnancy, the mother’s immune system undergoes significant changes. Different adaptation processes take place at various stages of pregnancy, involving both the innate and acquired immune responses. These changes are aimed at enabling the growth and development of the allogeneic fetus. However, in the event of encountering a pathogen, such as the influenza virus, the altered immune system may produce an inadequate response, potentially leading to complications in fetal development. The consequences of this scenario may depend on several factors, including the duration of pregnancy and the level of pre-pregnancy immunity [1].

Neuronal autoimmunity caused by infection may be involved in some acute psychoses, and it also plays a role in maternal transmission. The transfer of pathogenic antibodies from mother to fetus has long been considered a potential mechanism for the development of autism spectrum disorder and, to a lesser extent, schizophrenia [89]. While this concept is not formally part of the maternal immune activation model, recent experiments in animal models have made some progress in recapitulating neurodevelopmental phenotypes in immunization paradigms where maternal antibodies are transferred to the offspring, resulting in neuropathological and behavioral abnormalities [90,91,92]. Additionally, a study by Coutinho et al. [91] found that antibodies to the NMDA receptor (NMDAR) were more common in mothers of children with neurodevelopmental disorders who themselves subsequently developed psychosis.

A recent animal study demonstrated that the transfer of recombinant NMDAR NR1 antibodies from mother to fetus at levels that did not affect the behavior of the pregnant mother resulted in impaired neurodevelopmental reflexes, decreased anxiety, motor hyperactivity, and impaired sensorimotor gating. The latter two characteristics were considered to have psychosis-like phenotypes [93].

The absence of a role for the virus’ external antigens in the development of neurodegenerative changes through the production of autoantibodies is indirectly supported by extensive research on the impact of vaccinating pregnant women on autism spectrum disorders in their children [94]. The study demonstrates no association between maternal vaccination during pregnancy and the occurrence of such disorders. However, it is important to note that the study does not specify the types of vaccines (live, inactivated, or subunit) administered to pregnant women. Moreover, it is unlikely that proteins from neurotropic strains were used as the basis for the vaccine.

## 5. Features of the Course of Neurotropic Influenza in Animal Models

The studies by Fatemi et al. [95] highlight the importance of using a model of prenatal viral infection and detecting changes depending on the timing of infection to study neurodevelopmental disorders of the offspring, particularly autism and schizophrenia. Their research demonstrated that infection of pregnant mice with a mouse-adapted human influenza virus (A/NWS/33 H1N1) on embryonic days 7, 9, 16, and 18 (E7, E9, E16, and E18, respectively) resulted in abnormal brain gene expression, altered brain structure, neurochemical changes, and behavioral disturbances in the offspring of exposed mice. These findings are consistent with several biochemical, structural, and behavioral brain measures observed in patients with schizophrenia or autism.

The microarray analyses conducted in the study by Fatemi et al. revealed changes in gene expression that depended on the timing of prenatal viral infection. In Balb/c mice infected at E9, significant changes were observed in 205 genes at postnatal day 35 (P35) and 50 genes at P56 in the cerebellum of the offspring of exposed mice compared with controls. There was also a more pronounced effect of prenatal viral infection on cerebellar gene expression at E16 in C57BL/6J mice, with 98 genes at P0, 219 at P14, and 653 at P56 showing altered expression. Infection at E18 resulted in changes in 157 genes at P0, 16 genes at P14, and 96 genes at P56. Similar patterns of changes in gene expression were also observed in the prefrontal cortex and hippocampus of the offspring of exposed mice [95].

To understand the potential impact of influenza virus exposure on offspring brain development and function, as well as the potential future development of schizophrenia. two models (short- and long-term maternal exposure to a neurotropic strain of influenza A virus) were used [96]. The pregnant mice were challenged with influenza A/WSN/33 virus on 14 and 17 days of pregnancy. Viral RNA was found in fetuses’ brain three days after birth. Interestingly, depending on the dose, fetuses could not demonstrate any symptoms of disease and survived or died early [41]. The expression of gene studies performed on the 90th postnatal day showed the elevated level of several genes (encoding RING protein ring 1B, neuroleukin, and fibroblast growth factor 5). These results suggest that maternal infection may cause changes in gene expression in the brain that appear only when the offspring reaches early adulthood. Although the mice were tested in learning and memory tasks in which the hippocampus plays an important role, no differences were found.

Several studies [9,10,97] demonstrated that influenza virus infection using the NWSN/33 strain on day 9 of pregnancy can lead to behavioral disturbances and neurochemical dysregulation during puberty in exposed offspring. However, it is important to note that the virus strain used by Fatemi and colleagues did not enter the brain but caused severe disease behavior in pregnant mice. In contrast, the study by Simret Beraki et al. [96] found that virus-infected mice did not show any obvious signs of disease, such as weight loss, and influenza infection had no negative effect on offspring size.

Moreover, similar behavioral deficits were observed with injections of the synthetic cytokine-releasing polyI:C in other studies, suggesting that the behavioral effects on the offspring are believed to be caused by the maternal inflammatory response rather than by the virus itself [10,96].

## 6. Other Factors

A study [98] also explored vascular disorders linked to inflammation, including those that can lead to elevated blood pressure during viral infections. Additionally, it references research indicating that elevated blood pressure during pregnancy, unrelated to viral infection, is also associated with an increased risk of schizophrenia, depression, and anxiety in offspring.

Similar to other viruses, like Zika virus and cytomegalovirus, in the case of influenza A, the activation of proinflammatory cytokines (IL-6, IL-1b, TNF-a, and IFN-b) in the mother can induce neuroinflammation in the fetus. The expression of these factors can be triggered, in part, by the human leukocyte antigen DRB14. Additionally, hypoxia likely plays a role in neurodegeneration, as increased expression of hypoxic-inducible factor-1a (HIF-1a) is observed during inflammation. Moreover, maternal viral infection has been shown to lead to a reduction in the volume of the prefrontal, frontal, cingulate, insular, parietal, and temporo-auditory cortices in some cases, which can result in disturbances in exploratory behavior and social interaction in adulthood [99].

In a study [100] using a mouse model, it was demonstrated that moderately pathogenic influenza A during pregnancy results in inflammation in the placenta but does not impact the fetal brain. In cases of moderate disease, the role of IL-17A, which can modulate the inflammatory response and indirectly regulate the composition and function of the maternal microbiome in the intestine, is not significant, unlike in severe disease.

When studying animal models of neurodegeneration in offspring, it is important to consider factors that are sometimes overlooked, such as the interaction of pathogens and models of pathogens with components of the microbiome in the model animals [101].

A meta-analysis [102] examined research on neurodegeneration in offspring resulting from respiratory infections in mothers during pregnancy. According to the analysis, the authors propose that environmental conditions have a greater influence on neurodevelopment than the course of respiratory viral infections during pregnancy. However, they acknowledge potential abnormalities in early motor, behavioral, and socio-emotional areas in the presence of maternal infection during pregnancy and emphasize the need for more high-quality studies in the literature to draw definitive conclusions.

## 7. Prospects

Influenza infection during pregnancy presents a complex and clinically important issue. Understanding the mechanisms of interaction between the mother, the virus, and the immune system, at both systemic and local levels, is crucial for enhancing prevention and treatment strategies and warrants further investigation [1]. It is important not to overlook the identification and examination of the influence of other factors on the development of pregnancy complications, including those of a neuropsychiatric nature, for which high-quality epidemiological studies are necessary [12].

The development of better animal models to more accurately replicate the morbidity and mortality observed in human infections is crucial for advancing the study of the impact of infectious diseases during human pregnancy. Using animal, including gene-specific, models, researchers can better understand the role of genetic variation during influenza infection, particularly by identifying key polymorphisms, and this also helps identify at-risk groups and new targets for therapeutic interventions and vaccines [1,103]. As a result, research in this area is of growing interest and holds significant promise for advancing our understanding of the effects of infectious diseases during pregnancy.

In terms of future studies, investigating the relationship between infection and innate and adaptive immune responses in schizophrenia using animal models and large-scale serological studies in patients at different stages of the disease will be beneficial. Standardized and more sensitive testing technologies, including improved non-invasive methods, are required to assess central neuroinflammation in humans and animals [104,105]. Additionally, the development of next-generation genetic, immunological, and bioinformatics technologies may shed light on the relationship between influenza and psychosis [44].

It is evident that numerous factors can influence the development of neurological complications in offspring due to maternal influenza infection. Primarily, this effect is directly caused by the pathogen and the mother’s immune response to the infection. It is important to consider that pregnant women comprise a special group with limited options for therapeutic drug use. The establishment of appropriate models to study the mechanisms of neurological complications resulting from influenza in offspring will likely lead to the identification of potential therapeutic targets to mitigate the risk of complications. However, currently, the only way to protect both the mother and her offspring from illness and serious complications is through influenza vaccination, which is also permitted during pregnancy.
